# A 29-mRNA host-response classifier identifies bacterial infections following liver transplantation – a pilot study

**DOI:** 10.1007/s00423-024-03373-1

**Published:** 2024-06-12

**Authors:** Amelie Halder, Oliver Liesenfeld, Natalie Whitfield, Florian Uhle, Judith Schenz, Arianeb Mehrabi, Felix C. F. Schmitt, Markus A. Weigand, Sebastian O. Decker

**Affiliations:** 1 Heidelberg University, Medical Faculty Heidelberg, Department of Anesthesiology, Im Neuenheimer Feld 420, 69120 Heidelberg, Germany; 2 Inflammatix, Sunnyvale, CA USA; 3 Heidelberg University, Medical Faculty Heidelberg, Department of General, Visceral & Transplantation Surgery, Neuenheimer Feld 420, 69120 Heidelberg, Germany

**Keywords:** Liver transplantation, 29-mRNA host response, Bacterial infection, Immunosuppression

## Abstract

**Purpose:**

Infections are common complications in patients following liver transplantation (LTX). The early diagnosis and prognosis of these infections is an unmet medical need even when using routine biomarkers such as C-reactive protein (CRP) and procalcitonin (PCT). Therefore, new approaches are necessary.

**Methods:**

In a prospective, observational pilot study, we monitored 30 consecutive patients daily between days 0 and 13 following LTX using the 29-mRNA host classifier IMX-BVN-3b that determine the likelihood of bacterial infections and viral infections. True infection status was determined using clinical adjudication. Results were compared to the accuracy of CRP and PCT for patients with and without bacterial infection due to clinical adjudication.

**Results:**

Clinical adjudication confirmed bacterial infections in 10 and fungal infections in 2 patients. 20 patients stayed non-infected until day 13 post-LTX. IMX-BVN-3b bacterial scores were increased directly following LTX and decreased until day four in all patients. Bacterial IMX-BVN-3b scores detected bacterial infections in 9 out of 10 patients. PCT concentrations did not differ between patients with or without bacterial, whereas CRP was elevated in all patients with significantly higher levels in patients with bacterial infections.

**Conclusion:**

The 29-mRNA host classifier IMX-BVN-3b identified bacterial infections in post-LTX patients and did so earlier than routine biomarkers. While our pilot study holds promise future studies will determine whether these classifiers may help to identify post-LTX infections earlier and improve patient management.

**Clinical trial notation:**

German Clinical Trials Register: DRKS00023236, Registered 07 October 2020, https://drks.de/search/en/trial/DRKS00023236

**Supplementary Information:**

The online version contains supplementary material available at 10.1007/s00423-024-03373-1.

## Introduction

In patients suffering from end-stage liver disease (ESLD), liver transplantation (LTX) is a well-established curative therapeutic option [1, 2]. The outcomes of these patients have significantly improved by applying optimized posttransplant care. A key complication in these patients are severe infections, which may lead to transplant failure and/or increased mortality [3]. Most infections are of bacterial etiology and occur within the first four weeks following LTX [4, 5]. Routinely used biomarkers (such as C-reactive protein (CRP) and procalcitonin (PCT)) have limited specificity and sensitivity [6–8]. Direct detection methods, such as microbiological cultures and/or molecular methods, can be insensitive [9–11]. Therefore, new diagnostics are needed to allow for early and accurate detection of infections and initiation of early antimicrobial therapy.

The transcriptomic classifier IMX-BVN-3b has been reported to allow early detection of bacterial and viral infections [12]. IMX-BVN-3b is based on the expression of 29 host-response messenger RNA (mRNA) and is interpreted by a machine-learning algorithm [13]. The accuracy of this classifiers has been demonstrated in critically ill surgical and medical ICU patients treated in emergency departments [12, 14–16]. The accuracy for the diagnosis and prognosis of infections in transplant patients has not yet been determined.

In the present study we investigated the accuracy of the IMX-BVN-3bclassifiers to diagnose and prognose infections in post-LTX patients in comparison to routinely used biomarkers for the detection of bacterial and viral infections. Moreover, the kinetics of classifier results were determined between day 0 and 13 post-LTX.

## Materials and methods

### Study design

The observational single-center pilot study was approved by the local ethics committee (Ethics Committee of the Medical Faculty of Heidelberg, Trial Code No. S-693/2020 / German Clinical Trials Register: DRKS00023236) and conducted in the surgical unit of the Heidelberg University Hospital, Germany between July 2021 and February 2023. All study participants gave written informed consent. In total, 30 patients suffering from ESLD undergoing orthotopic LTX were enrolled in this study, representing the only inclusion criterion. Missing written consent and age below 18 years were defined as exclusion criteria. The treatment of patients was based on the Heidelberg Manual for LTX [17]. Blood was collected in PAXgene® Blood RNA tubes (PreAnalytix, Hombrechtikon, Switzerland) daily between days 0 and 13 post-LTX for testing of IMX-BVN-3b and IMX-SEV-3b classifiers; plasma samples were collected at the same time points post-LTX to determine CRP and PCT concentrations. Relevant baseline data (demographic data, primary site of infection), clinical data (disease severity scores, such as Simplified Acute Physiology (SAPS II)-score, Sequential Organ Failure Assessment (SOFA)-score and Acute Physiology Health Evaluation (APACHE II)-score, surgical procedures, antifungal therapy, outcome parameters) and routine infection parameters (leukocytes, C-reactive protein (CRP), procalcitonin (PCT), body temperature), according to the standardized central laboratory protocols, were also collected.

The primary endpoint of this study was the accuracy of the IMX-BVN-3b for the detection of bacterial and viral infections following LTX as determined by clinical adjudication. Secondary endpoints included a) the kinetics of IMX-BVN-3b post-LTX and b) the comparison of IMX-BVN-3b accuracy to biomarkers CRP and PCT.

**Fig. 1:**
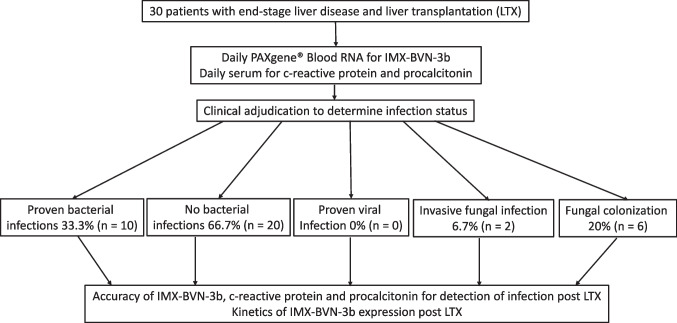
Flowchart of included patients. Abbreviations: LTX, liver transplantation

A detailed study flowchart is shown in Fig. 1. The study was performed according to the Strobe statement (Supplementary Material 1).

### Clinical adjudication

The reference standard was determined by a chart review to establish the true infection status for the presence or absence of a bacterial, viral and/or other infection. The expert panel consisted of two physicians trained in infectious diseases who classified the patients for bacterial, viral and fungal infections according to previously published criteria[18, 19] and in accordance to Centers for Disease Control and Prevention (CDC) /National Healthcare Safety Network (NHSN) criteria for bacterial infections as well as European Organization for Research and Treatment of Cancer (EORTC) criteria for invasive fungal infections [20, 21]. They used medical history, physical examination findings, all available laboratory, including CRP and PCT, microbiological and imaging data, and the discharge report or treatment data. The expert panel was blinded to the IMX-BVN-3b results.

### Blood sample collection in PAXgene tube and amplification of target genes

2.5 ml of whole blood were obtained daily using the PAXgene® Blood RNA tube. The tubes were frozen at -80 °C. Pseudonymized samples were shipped in batches to the Inflammatix laboratory (Sunnyvale, CA, USA) for gene amplification and IMX-BVN-3b classifier processing as previously described [12]. Operators at Inflammatix were blinded to any other study results.

### IMX-BVN-3b classifiers

The machine learning algorithms IMX-BVN-3b read and interpreted results of amplification for the 29 host mRNAs [22]. Results of the IMX-BVN-3b classifier were provided as numerical scores that fall into one of five interpretation bands for the likelihood of a bacterial infection, and into one of five interpretation bands for the likelihood of a viral infection. Predefined thresholds are used to categorize results into the Very low, Low, Moderate, High, or Very high bacterial and viral interpretation bands. Classifier results were provided to the Heidelberg study team for statistical analysis.

### Statistical analysis

All data were entered into an electronic database (Excel 2021; Microsoft Corp, Redmond, USA) and analyzed using SPSS software (Version 28.0; SPSS, Inc., Chicago, USA). Figures were drawn with GraphPad Prism 10 (GraphPad Software, La Jolla, USA), SPSS software and assembled with the presentation software PowerPoint 2021 (Microsoft Corp, Redmond, USA). Categorical data were shown as absolute and relative frequencies. Quantitative data were presented as median with quartiles. The Kolmogorov–Smirnov test was used to check for normal distribution. Due to non-normally distributed data, non-parametric methods for evaluation were used (Chi-square test for categorical data, Mann–Whitney U test for continuous data). Receiver-Operating characteristics were used to assess discrimination. Spearman correlation was used to detect significant correlations between different scores. We introduced virtual timepoints (V) for the first bacterial infection to normalize the analysis. Furthermore, infected patients were compared to a comparison group including non-infected age- and sex-matched patients. Moreover patients were classified in bands according to predefined cut off values, regarding IMX-BVN-3b [12, 14], PCT [23, 24] and CRP [25–27] for prediction of bacterial infections and calculation of likelihood ratios. A *p*-value < 0.05 was considered statistically significant.

## Results

### Patient characteristics and microbiological findings

Between day 0 to day 13 post LTX, 10 (33.3%) of 30 patients developed a bacterial infection as determined by clinical adjudication whereas 20 patients showed no signs of bacterial infections; there were no viral infections during the observation period. Anatomical sites of bacterial infection were intraabdominal (*n* = 6), respiratory (pneumonia, *n* = 1) and urinary tract (*n* = 1); two patients developed bacteriemia with positive blood cultures. Invasive fungal infections were detected in two (6.7%) patients; one with *Aspergillus fumigatus* in the lung and one with *Candida albicans* in the abdomen, 6 (20%) patients were colonized with fungal pathogens (*Candida* species). General clinical characteristics of patients are presented in Table 1, clinical characteristics of ICU and hospital stay are shown in Table 2. Patients with or without bacterial infection did not differ significantly with regard to underlying diseases, immunosuppressive medications or other clinical characteristics (Table 1)**.** Patients with bacterial infections had significantly longer hospital and ICU length of stay; they also showed more significant blood loss during surgery and a significantly higher proportion of patients with bacterial infections received platelets (Table 2).

**Table 1:** Patients’ characteristics

Parameter	Unit	All patients (*n* = 30)	With bacterial infection (*n* = 10)	Without bacterial infection (*n* = 20)	*p* *for patients without bacterial infection vs. with bacterial infection*
male		17 (56.7%)	8 (80.0%)	9 (45.0%)	0.074
age	(years)	56 (47–61)	55.5 (47.25–62.00)	56 (45.75–61.25)	0.779
BMI	(kg/m^2^)	25.32 (22.95–29.61)	24.84 (23.66–27.77)	24.47 (24.48–26.75)	0.948
MELD-Score		26 (22–31)	25 (24–27)	26 (22–31)	0.941
Causes of liver cirrhosis					
Alcohol		11 (36.7%)	3 (30.0%)	8 (40.0%)	0.452
Hepatitis B		1 (3.3%)	1 (10.0%)	0 (0.0%)	0.333
Hepatitis C		3 (10.0%)	1 (10.0%)	2 (10.0%)	0.749
HCC		9 (30.0%)	3 (30.0%)	6 (30.0%)	0.306
PSC		3 (10.0%)	0 (0.0%)	3 (15.0%)	0.281
PBC		1 (3.3%)	1 (10.0%)	0 (4.3%)	0.333
NASH		3 (10.0%)	1 (10.0%)	2 (10.0%)	0.749
Need for catecholamines before LTX		3 (10.0%)	1 (10.0%)	2 (10.0%)	0.749
NYHA 0-I		29 (96.7%)	10 (100%)	19 (95.0%)	0.667
Diabetes mellitus		10 (33.3%)	0 (0.0%)	10 (50.0%)	0.177
Arterial hypertension		13 (43.3%)	4 (40.0%)	9 (45.0%)	0.554
Coronary heart disease		4 (13.3%)	1 (10.0%)	3 (15.0%)	0.593
Chronic obstructive lung disease		5 (16.7%)	2 (20.0%)	3 (15.0%)	0.551
Smoker		12 (40.0%)	4 (40.0%)	8 (40.0%)	0.656
CKD		10 (33.3%	4 (40.0%)	6 (30%)	0.440
Pre-existing ARF		5 (16.7%)	2 (20.0%)	3 (15.0%)	0.551
Pre-existing thrombosis		5 (16.7%)	3 (30.0%)	2 (10.0%)	0.191
Neurological disorder		17 (56.7%)	5 (50.0%)	12 (60.0%)	0.446
High-urgency		1 (3.3%)	0 (0.0%)	1 (5.0%)	0.667
Re-LTX		2 (6.7%)	2 (20.0%)	0 (0.0%)	0.102
Immunosuppressive medication					
Cortisosteroids		30 (100%)	10 (100%)	20 (100%)	–-
Mycophenolat mofetil		30 (100%)	10 (100%)	20 (100%)	–-
Ciclosporin		0 (0.0%)	0 (0.0%)	0 (0.0%)	–-
Tacrolimus		30 (100%)	10 (100%)	20 (100%)	–-

Data are presented either as number (with the corresponding percentage value) or as median (with accompanying quartiles (Q1–Q3)

Legends: *BMI* = Body Mass Index, *MELD* = Model of end-stage liver disease, *HCC* = hepatocellular carcinoma, *PSC* = primary sclerosing cholangitis, *PBC* = Primary Biliary Cirrhosis, *NASH* = Non-alcoholic Fatty Liver Disease, *NYHA* = New York Heart Association, *CKD* = Chronic kidney disease, *ARF* = acute renal failure, *LTX* = liver transplantation.Concerning symbolism and higher orders of significance: *p* < 0.05: *

**Table 2:** Details of the ICU and hospital stay

Parameter	Unit	All patients (*n* = 30)	With bacterial infection (*n* = 10)	Without bacterial infection (*n* = 20)	*p* *for patients without bacterial infection vs. with bacterial infection*
APACHE II^+^		16.0 (13.3–21.0)	16.5 (15.3–27.0)	16.0 (12.0–19.5)	0.183
SOFA^+^		6.5 (5.0–9.8)	9.5 (6.8–10.8)	5.5 (3.0–7.3)	0.024*
SAPS^+^		31.0 (21.0–42.5)	39.0 (33.8–55.8)	23.5 (15.8–38.3)	0.039*
Time of mechanical Ventilation	(days)	0 (0–2.8)	3.0 (2.0–4.0)	0.5 (0–1)	0.003**
Tracheostomy		1 (5.0%)	1 (10.0%)	0 (0.0%)	0.333
Hospital stay before LTX	(days)	0 (0–8.3)	0.0 (0.0–20.3)	0.0 (0.0–2.8)	0.605
ICU stay	(days)	17.0 (13.3–33.0)	39.5 (16.3–12.8)	15 (11.8–70.0)	0.031*
Hospital stay	(days)	32.0 (25.0–62.0)	71.5 (33.0–87.0)	30 (25.0–38.0)	0.035*
90-day survival		29 (96.7%)	9 (90.0%)	20 (100%)	0.333
28-day survival		30 (100%)	10 (100%)	20 (100%)	––
ALF after LTX		1 (5.0%)	1 (10.0%)	0 (0.0%)	0.333
ARF after LTX		24 (80.0%)	8 (80.0%)	16 (80.0%)	0.674
Dialysis Directly after LTX In time course		3 (10%) 11 (36.7%)	2 (20.0%) 5 (50.0%)	1 (5.0%) 6 (30.0%)	0.251 0.250
BDA		2 (6.7%)	1 (10%)	1 (5.0%)	0.563
Duration of surgery	(min)	345 (330–390)	375.0 (330.0–412.5)	330.0 (322.5–360.0)	0.307
Intraoperative blood loss	(L)	2.5 (1.7–4.7)	5.0 (3.0–13.0)	2.5 (1.0–3.2)	0.010*
Administration of					
PRBC	(n.o.p.)	24 (80.0%)	10 (100%)	14 (70.0%)	0.065
FFP	(n.o.p.)	28 (93.3%)	10 (100%)	18 (90.0%)	0.437
Platelets	(n.o.p.)	16 (53.3%)	8 (80.0%)	8 (40.0%)	0.045*
Rejection		2 (6.7%)	2 (20.0%)	0 (0.0%)	0.103

Data are presented either as number (with the corresponding percentage value) or as median (with accompanying quartiles (Q1–Q3)

Legends: APACHE II–Score = Acute Physiology And Chronic Health Evaluation score, *SOFA* = Sequential Organ Failure Assessment score, *SAPS* = Simplified Acute Physiology score, *ICU* = intensive care unit, *LTX* = liver transplantation, *ARF* = acute renal failure, *ALF* = acute liver failure, *BDA* = biliodigestive anastomosis, n.o.p. = number of patients, PRBC = packed red blood cells, *FFP* = fresh frozen plasma,^+^ calculated at the first day after LTX. Concerning symbolism and higher orders of significance: *p* < 0.05: *, *p* < 0.01**, *p* < 0.001***

### IMX-BVN-3b for the diagnosis of bacterial infections

**Fig. 2:**
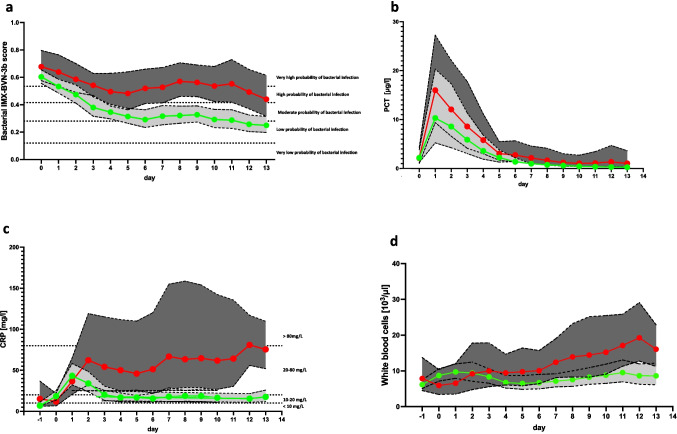
Bacterial IMX-BVN-3b scores and inflammatory markers in patients following liver transplantation. (**a**) Bacterial IMX-BVN-3b scores were measured in patients following liver transplantation. Patients were divided according to the occurrence of a proven bacterial infection within the first 14 days following LTX. Bacterial IMX-BVN-3b scores are presented for unobtrusive patients as green line combined with light grey area and for patients with bacterial infections as red line combined with a dark grey area. Plasma samples were collected immediately following liver transplantation (d0), and the days afterwards until day 13 as indicated. Data are presented as median with 95% Confidence interval as borders of the areas. (**b**) Procalcitonin (PCT) was measured in patients following liver transplantation. Patients were divided according to the occurrence of a proven bacterial infection within the first 14 days following LTX. PCT levels are presented for unobtrusive patients as green line combined with light grey area and for patients with bacterial infections as red line combined with a dark grey area. Plasma samples were collected immediately following liver transplantation (d0), and the days afterwards until day 13 as indicated. Data are presented as median with 95% Confidence interval as borders of the areas. (**c**) C-reactive protein (CRP) was measured in patients following liver transplantation. Patients were divided according to the occurrence of a proven bacterial infection within the first 14 days following LTX. CRP levels are presented for unobtrusive patients as green line combined with light grey area and for patients with bacterial infections as red line combined with a dark grey area. Plasma samples were collected immediately following liver transplantation (d0), and the days afterwards until day 13 as indicated. Data are presented as median with 95% Confidence interval as borders of the areas. (**d**) White blood cell counts were measured in patients following liver transplantation. Patients were divided according to the occurrence of a proven bacterial infection within the first 14 days following LTX. White blood cell counts are presented for unobtrusive patients as green line combined with light grey area and for patients with bacterial infections as red line combined with a dark grey area. Plasma samples were collected immediately following liver transplantation (d0), and the days afterwards until day 13 as indicated. Data are presented as median with 95% Confidence interval as borders of the areas

IMX-BVN-3b scores were at high scores in all patients directly following LTX (Fig. 2a). In patients with bacterial infections, scores were significantly higher and stayed high compared to noninfected patients (Supplementary Fig. 1), in whom scores decreased over time. In contrast, the routinely used infection parameters PCT, CRP and white blood cell count showed limited value in the detection of bacterial infections (Fig. 2b-d and Supplementary Figs. 2a-c). PCT levels were highly increased starting from day one following LTX in all patients and declined in the days after, independently from the occurrence of bacterial infections (Fig. 2b and Supplementary Fig. 2a). CRP levels were increased in all patients starting from day one following LTX. In uninfected patients CRP declined in the days afterwards, whereas in patients with proven bacterial infection CRP showed significant elevated levels. Nevertheless, all patients presented with values indicating an inflammatory reaction using the cut-offs for uninfected patients (Fig. 2c and Supplementary Fig. 2b). White blood cell count showed undulating values in all patients with a trend to lower values in patients without bacterial infections compared to patients with bacterial infections without showing relevant significant differences (Fig. 2d and Supplementary Fig. 2c).

These findings could be proven within the likelihood analysis of IMX-BVN-3b, CRP and PCT (Supplementary Table 1 A to C), indicating the best performance for IMX-BVN-3b around day 6 to 9 following LTX, correlating with the fact that most bacterial infections occurred during this period.

**Fig. 3:**
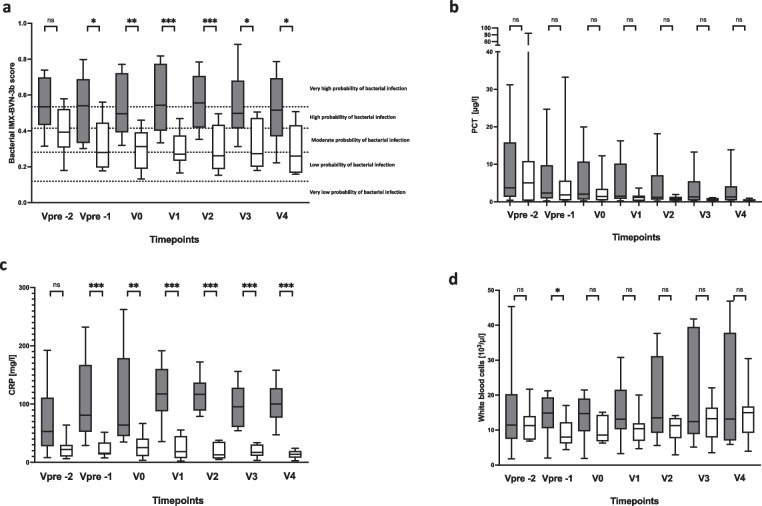
Bacterial IMX-BVN-3b scores and inflammatory markers in patients following liver transplantation adjusted to the timepoint of the bacterial infection. (**a**) Bacterial IMX-BVN-3b scores were measured in patients following liver transplantation. Measurements in patients with bacterial infection were adjusted to the timepoint of appearance (grey box) and then compared to non-infected patients (white box). In patients with a bacterial infection, new timepoints were created by matching them to the first time of bacterial infection, whereas the control group without bacterial infection was created by matching them in an age- and sex-related manner to the same timepoints of patients with a bacterial infection. The following virtual timepoints were created: the first measurement two days before the first bacterial infection (preV-2), the first measurement two days before the first bacterial infection (preV-1), the plasma level at the time of bacterial infection (V0) and the next measured plasma levels on the days afterwards (V1-V4). Data in box plots are given as median, 25th percentile, and 75th percentile, with the 10th and 90th percentile at the end of the whiskers. Concerning symbolism and higher orders of significance: *p* < 0.05: *; *p* < 0.01: **; *p* > 0.001: ***Mann–Whitney U Test. (**b**) Procalcitonin (PCT) was measured in patients following liver transplantation. Measurements in patients with bacterial infection were adjusted to the timepoint of appearance (grey box) and then compared to non-infected patients (white box). In patients with a bacterial infection, new timepoints were created by matching them to the first time of bacterial infection, whereas the control group without bacterial infection was created by matching them in an age- and sex-related manner to the same timepoints of patients with a bacterial infection. The following virtual timepoints were created: the first measurement two days before the first bacterial infection (preV-2), the first measurement two days before the first bacterial infection (preV-1), the plasma level at the time of bacterial infection (V0) and the next measured plasma levels on the days afterwards (V1-V4). Data in box plots are given as median, 25th percentile, and 75th percentile, with the 10th and 90th percentile at the end of the whiskers. Concerning symbolism and higher orders of significance: *p* < 0.05: *; *p* < 0.01: **; *p* > 0.001: ***Mann–Whitney U Test. (**c**) C-reactive protein (CRP) was measured in patients following liver transplantation. Measurements in patients with bacterial infection were adjusted to the timepoint of appearance (grey box) and then compared to non-infected patients (white box). In patients with a bacterial infection, new timepoints were created by matching them to the first time of bacterial infection, whereas the control group without bacterial infection was created by matching them in an age- and sex-related manner to the same timepoints of patients with a bacterial infection. The following virtual timepoints were created: the first measurement two days before the first bacterial infection (preV-2), the first measurement two days before the first bacterial infection (preV-1), the plasma level at the time of bacterial infection (V0) and the next measured plasma levels on the days afterwards (V1-V4). Data in box plots are given as median, 25th percentile, and 75th percentile, with the 10th and 90th percentile at the end of the whiskers. Concerning symbolism and higher orders of significance: *p* < 0.05: *; *p* < 0.01: **; *p* > 0.001: ***Mann–Whitney U Test. (**d**) White blood cell counts were measured in patients following liver transplantation. Measurements in patients with bacterial infection were adjusted to the timepoint of appearance (grey box) and then compared to non-infected patients (white box). In patients with a bacterial infection, new timepoints were created by matching them to the first time of bacterial infection, whereas the control group without bacterial infection was created by matching them in an age- and sex-related manner to the same timepoints of patients with a bacterial infection. The following virtual timepoints were created: the first measurement two days before the first bacterial infection (preV-2), the first measurement two days before the first bacterial infection (preV-1), the plasma level at the time of bacterial infection (V0) and the next measured plasma levels on the days afterwards (V1-V4). Data in box plots are given as median, 25th percentile, and 75th percentile, with the 10th and 90th percentile at the end of the whiskers. Concerning symbolism and higher orders of significance: *p* < 0.05: *; *p* < 0.01: **; *p* > 0.001: ***Mann–Whitney U Test

In a subsequent analysis, focusing on the timepoint of the bacterial infection, IMX-BVN-3b was also able to correctly identify patients with bacterial infections at least one day before the clinical diagnosis (Fig. 3a and Supplementary Fig. 3a), which could be verified by a ROC-analysis (Supplementary Fig. 3b). In contrast, PCT was unable to significantly differentiate between patients with and without bacterial infections (Fig. 3b). On the other hand, CRP showed a comparable course to IMX-BVN-3b for the diagnosis of bacterial infection, when focusing on the timepoint of infection (Fig. 3c). Regarding the white blood cell count, no significant differences could be observed in patients with or without bacterial infection at timepoint of the clinical diagnosis (Fig. 3d). These findings could be proven within the likelihood analysis of IMX-BVN-3b, CRP and PCT (Table 3 A to C). Moreover, in detailed analysis of IMX-BVN-3b curves of each single patient, nine of the 10 patients with adjudicated bacterial infection were correctly identified by the test (Supplementary Fig. 3a).

**Table 3.**
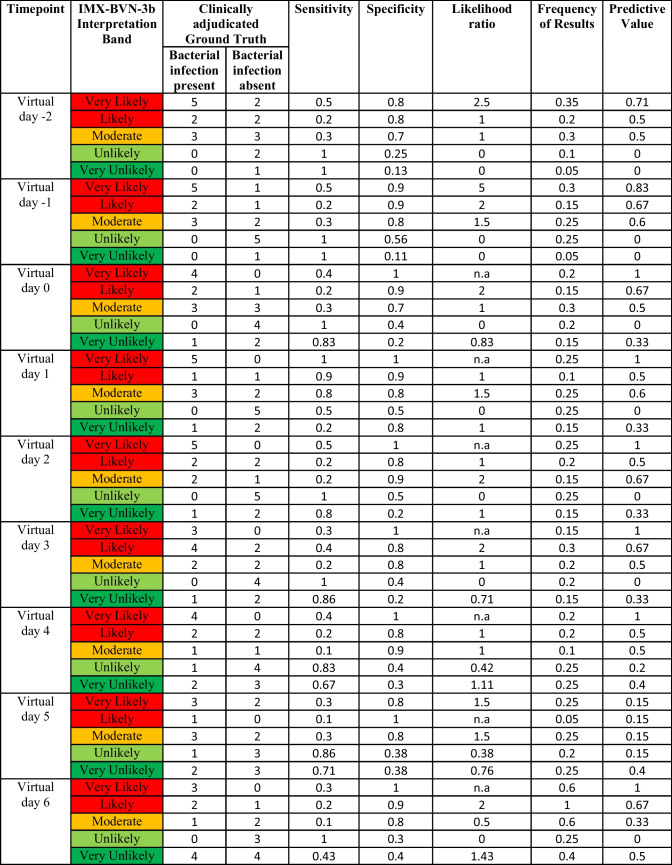

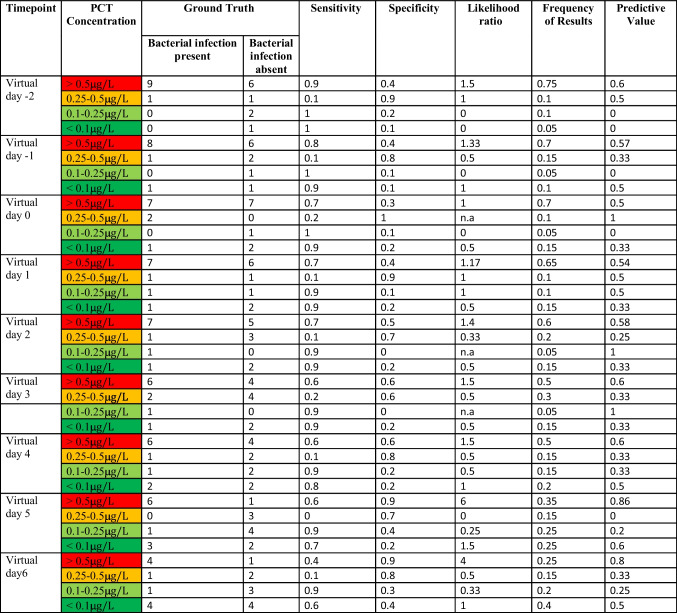

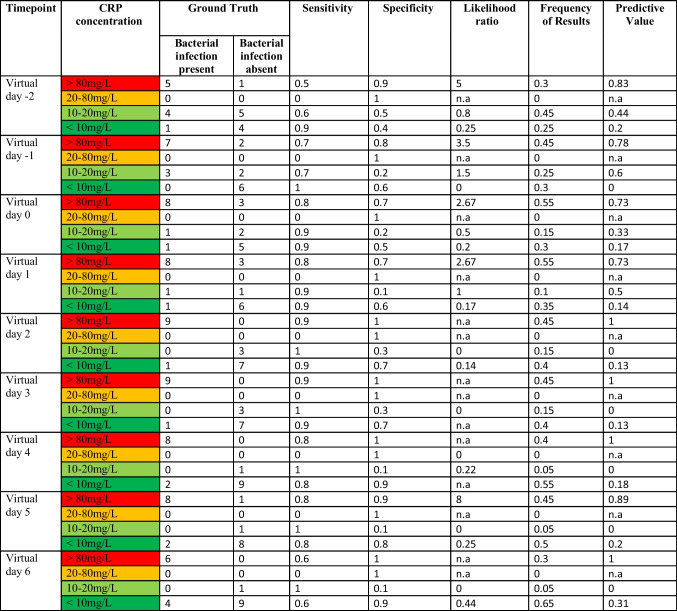
Accuracy of IMX-BVN-3b, procalcitonin and C-reactive protein for the diagnosis of bacterial infections at timepoint of the bacterial infection

### IMX-BVN-3b for the detection of viral infections following LTX

Clinical adjudication did not diagnose any viral infections. Viral IMX-BVN-3b scores did not increase during the observation period (Supplementary Fig. 4).

### IMX-BVN-3b in patients with fungal infections vs. fungal colonization

Clinical adjudication confirmed invasive fungal infections in two patients, whereas 6 patients were found to be colonized with fungi. IMX-BVN-3b bacterial and viral scores did not increase in patients with fungal infections. Bacterial and viral IMX-BVN-3b scores were similar in patients with fungal colonization compared to the uninfected patients (data not shown).

## Discussion

The present study shows the potential utility of the IMX-BVN-3b test for the earlier detection of bacterial infections following LTX and differentiation of uninfected patients.

Bacterial infections following LTX are common events due to the need of immunosuppression and surgical procedures, especially in the early period of post-transplant care [4, 5]. The routinely used culture diagnostics have long turnaround times until results are available and are associated with relevant weakness, due to false positive or negative results [28, 29]. Available molecular diagnostics approaches including polymerase-chain reaction (PCR) analysis, which is linked with limited number of detected pathogens or next-generation-sequencing methods have been tested in several study settings [19, 30], but are far away from being used as routine methods.

Plasma biomarkers, like CRP, PCT or white blood cell count are routinely used in daily clinical care but have relevant limitations [11, 31]. Therefore, new diagnostic approaches are needed, as time delays in the reliable diagnostic of bacterial infections in patients following LTX might result in increased morbidity and mortality of the affected patient [3].

The combined host response signature test IMX-BVN-3b is still under development by Inflammatix, Inc. The test is able to quantify 29 messenger RNA (mRNA) expressed in whole blood, which were derived from transcriptomic studies in patients with critical illness or sepsis [32–34]. Using a machine learning classifier, the prognostic performance in patients with sepsis or severe illness was optimized [35]. Nevertheless, this is also a limiting point, as the test has not been solely examined in patients following liver transplantation or other solid organ transplantation until now. Also, the test is not yet commercially available. Despite these limitations, already published data showed promising results of earlier, reliable diagnosis in critical ill patients [12, 16]. Patients following LTX are good comparators to patients with severe sepsis and maybe more in danger due to the need of immunosuppressive drugs to avoid rejection, which increases the risk of severe infections, with an inadequate response of the immune system, leading to a severe critical illness [4, 5]. As already described above, routinely used diagnostics methods, e.g., CRP or PCT are fraught with limitations, which has also been shown in our department [36]. Moreover, PCT levels were described to be increased directly after liver transplantation without a relevant source of infection, presumably due to a transfer from the donor [37, 38], rendering it in particular as a single parameter to be less usable in patients following LTX [39]. Therefore, the test that may be commercially available in the future that includes the IMX-BVN-3b classifier (or a future version of it) might be a promising new rapid (~ 30 min) diagnostic option [40]. As shown here in the presented study, bacterial IMX-BVN-3b scores are highly elevated in all patients directly following LTX, potentially signaling a severe bacterial infection, which might be due to a donor-received factor introduced during the transplantation from the donor organ, comparable to PCT. In contrast to PCT, bacterial IMX-BVN-3b scores dropped to low scores in patients without proven bacterial infection, whereas scores in patients with infections stayed high, indicative of the existing inflammation. Therefore, bacterial IMX-BVN-3b may be an additional option in routine care to optimize the diagnostic options in patients following LTX with comparable performance as shown in patients with sepsis [12, 15]. This point is highlighted by the fact, that adjusted to the timepoint of bacterial infection, bacterial IMX-BVN-3b showed high scores one day before the clinical diagnosis of the bacterial infections comparable to CRP.

Viral infections, like cytomegalovirus, in patients following LTX may also lead to increased morbidity and poor outcome of the affected patient as the virus can induce acute rejections [41, 42]. Most of the viral infections are due to a virus reactivation, despite antiviral prophylaxis, following first virus contact and long before transplantation due to persistence within the body which may lead to a delayed diagnosis [43–45]. Therefore, a fast and reliable diagnosis of such infections is necessary. In the present study, none of the patients showed signs of a viral infection. These findings were also proven by the viral IMX-BVN-3b score. Within the whole cohort, viral IMX-BVN-3b levels stayed at low levels, indicating no signs of a probable viral infection. Nevertheless, these findings should be reevaluated in a larger cohort, that includes patients exhibiting a viral infection post-LTX.

The fact, that IMX-BVN-3b was not able to differ between patients with fungal infection and without such an infection might be explained due to the underlying data base of the test, which was not developed for fungal infections. As this is a machine learning system [22], further development approaches might offer the option to detect fungal infections in patients following LTX as well as suffering by other diseases.

### Limitations

This study had several limitations. Due to the ongoing Covid-19 pandemic in 2020 and 2021 the planned number of 50 patients could not be reached as the rates of transplantation were reduced. Due to organizational and feasibility reasons, the recruitment was stopped after 30 patients. All planned laboratory measurements were carried out and revealed plausible results. Moreover, the observation period was limited to just the first 14 days following LTX which was combined with a long-term observation period to prove the outcome of each single patient. Therefore, infections at later timepoints than 14 days may have been missed. Enlarging the observation period could have led to an increased burden for each single patient due to the need for more blood samples as well as for the study teams to ensure the feasibility of the study. Nevertheless, the presented results are conclusive, but will need to be verified within a larger study in the future.

## Conclusions

This study showed for the first time the kinetics of the 29-mRNA classifier IMX-BVN-3b in the early post-surgery care of patients following LTX and offered promising results for the earlier detection of bacterial infections in this time period. Moreover, it may have additional value on the routinely used inflammatory markers (CRP, PCT) and microbiological diagnostics for treatment decision, especially in cases of difficult conditions and uncertainty of a need for treatment. The viral score of the 29-mRNA classifier was able to rule out viral infections following LTX.

### Supplementary Information

Below is the link to the electronic supplementary material.

Supplementary file1 (PDF 736 KB)

## Data Availability

The data presented in this study are available on request from the corresponding authors.

## Abbreviation

APACHE II: Acute Physiology Health Evaluation score
ARF: Acute renal failure
BDA: Biliodigestive anastomosis
BMI: Body Mass Index
CDC: Centers for Disease Control and Prevention
CKD: Chronic kidney disease
CRP: C-reactive protein
EORTC: European Organisation for Research and Treatment of Cancer
FFP: Fresh frozen plasma
HCC: Hepatocellular carcinoma,
ICU: Intensive care unit
LTX: Liver transplantation
MELD: Model of end-stage liver disease
mRNA: Messenger RNA
n.a: Not applicable
NASH: Non-alcoholic Fatty Liver Disease
NHSN: National Healthcare Safety Network
n.o.p.: Number of patients
NYHA: New York Heart Association
PBC: Primary Biliary Cirrhosis
PCR: Polymerase-chain reaction
PCT: Procalcitonin
PRBC: Packed red blood cells
PSC: Primary sclerosing cholangitis
SAPS II: Simplified Acute Physiology score,
SDC: Supplemental Digital Content
SOFA: Sequential Organ Failure Assessment score
V: Virtual timepoint

## Declarations

### Clinical trial notation

German Clinical Trials Register: DRKS00023236, Registered 07 October 2020, https://drks.de/search/en/trial/DRKS00023236
