# Supplementary material for: A 29-mRNA host-response classifier identifies bacterial infections following liver transplantation – a pilot study
Source: Langenbecks Arch Surg. 2024 Jun 12;409(1):185. doi: 10.1007/s00423-024-03373-1 (PMC11169022; doi:10.1007/s00423-024-03373-1)
Supplement: Supplementary file 1 — Supplementary file1 (PDF 736 KB) [file 423_2024_3373_MOESM1_ESM.pdf]

Supplementary Figure 1: Bacterial IMX-BVN-3b scores in patients following liver transplantation.

a

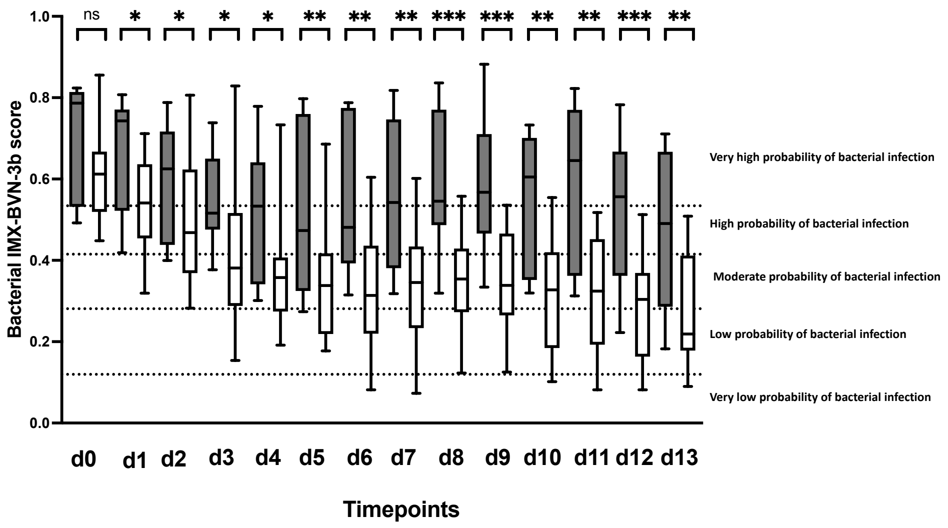

b

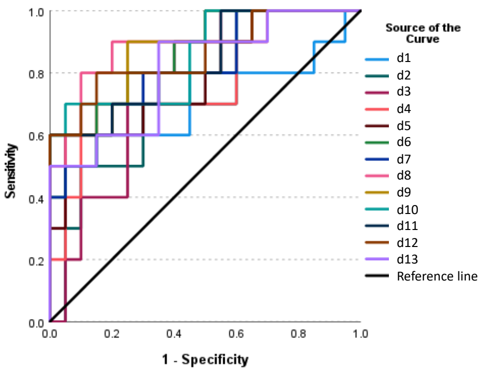

Timepoint	AUC	95% CI
d1	0.730	0.498 - 0.962
d2	0.725	0.534 - 0.916
d3	0.780	0.616 - 0.944
d4	0.765	0.573 - 0.957
d5	0.785	0.605 - 0.965
d6	0.850	0.710 - 0.990
d7	0.810	0.643 - 0.977
d8	0.900	0.780 - 1.020
d9	0.880	0.753 - 1.007
d10	0.855	0.704 - 1.006
d11	0.835	0.676 - 0.994
d12	0.860	0.706 - 1.014
d13	0.810	0.642 - 0.978

(a) Bacterial IMX-BVN-3b scores were measured in patients following liver transplantation. Patients were divided according to the occurrence of a proven bacterial infection within the first 14 days following LTX. Bacterial IMX-BVN-3b scores are presented for unobtrusive patients (white box) and patients with bacterial infection (grey box). Plasma samples were collected immediately following liver transplantation (d0), and the days afterwards until day 13 as indicated. Data in the box plots are given as the median, 25th percentile, and 75th percentile with the 10th as well as 90th percentile at the end of the whiskers. Concerning symbolism and higher orders of significance: $p < 0.05$: *; $p < 0.01$: **; $p > 0.001$: ***, Mann-Whitney U Test.

(b) Receiver operating characteristic (ROC) analysis with Bacterial IMX-BVN-3b scores in all participating patients at the indicated days with regard to the detection of bacterial infections. Patients who developed a proven bacterial infections following liver transplantation represented the target group, whereas unobtrusive served as controls for this ROC analysis.

Supplementary Figure 2: Plasmatic levels of procalcitonin (PCT), C-reactive protein (CRP) and white blood cell counts in patients following liver transplantation

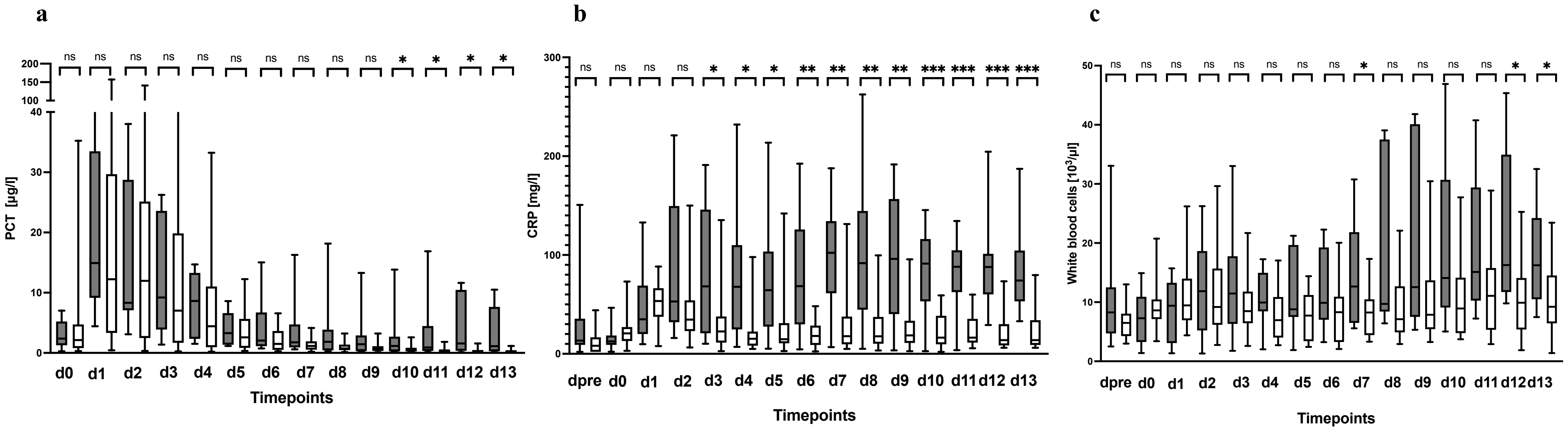

(a) Procalcitonin (PCT) was measured in patients following liver transplantation. Patients were divided according to the occurrence of a proven bacterial infection within the first 14 days following LTX. PCT levels are presented for unobtrusive patients (white box) and patients with bacterial infection (grey box). Plasma samples were collected immediately following liver transplantation (d0), and the days afterwards until day 13 as indicated. Data in the box plots are given as the median, 25th percentile, and 75th percentile with the 10th as well as 90th percentile at the end of the whiskers. Concerning symbolism and higher orders of significance: $p < 0.05$: *, Mann-Whitney U Test.

(b) C-reactive protein (CRP) was measured in patients following liver transplantation. Patients were divided according to the occurrence of a proven bacterial infection within the first 14 days following LTX. CRP levels are presented for unobtrusive patients (white box) and patients with bacterial infection (grey box). Plasma samples were collected immediately following liver transplantation (d0), and the days afterwards until day 13 as indicated. Dpre indicates the last measurement the day before LTX. Data in the box plots are given as the median, 25th percentile, and 75th percentile with the 10th as well as 90th percentile at the end of the whiskers. Concerning symbolism and higher orders of significance: $p < 0.05$: *, Mann-Whitney U Test.

(c) White blood cell counts were measured in patients following liver transplantation. Patients were divided according to the occurrence of a proven bacterial infection within the first 14 days following LTX. White blood cell counts are presented for unobtrusive patients (white box) and patients with bacterial infection (grey box). Plasma samples

were collected immediately following liver transplantation (d0), and the days afterwards until day 13 as indicated. Dpre indicates the last measurement the day before LTX. Data in the box plots are given as the median, 25th percentile, and 75th percentile with the 10th as well as 90th percentile at the end of the whiskers. Concerning symbolism and higher orders of significance: $p < 0.05$: *, Mann-Whitney U Test.

Supplementary Figure 3: Bacterial IMX-BVN-3b scores in patients following liver transplantation adjusted to the timepoint of the bacterial infection.

a

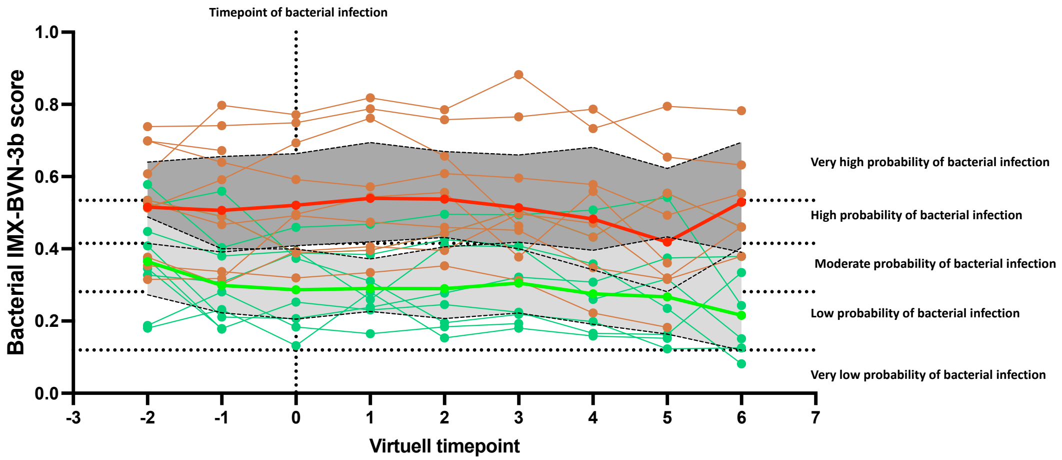

b

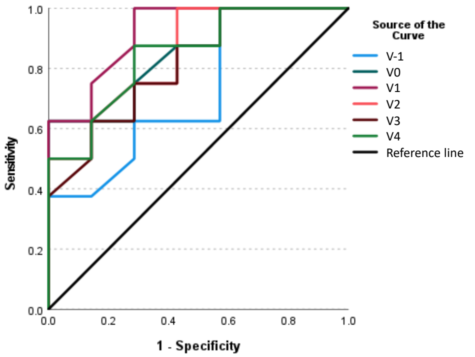

Timepoint	AUC	95%-CI
V-1	0.723	0.458 - 0.988
V0	0.848	0.651 - 1.045
V1	0.920	0.781 - 1.058
V2	0.848	0.651 - 1.045
V3	0.813	0.594 - 1.031
V4	0.848	0.651 - 1.045

(a) Bacterial IMX-BVN-3b scores were measured in patients following liver transplantation. Bacterial IMX-BVN-3b-scores are presented for unobtrusive patients as green line combined with light grey area and light green points for each single course and for patients with bacterial infections as red line combined with a dark grey area and orange points for each single course. In patients with a bacterial infection, new timepoints were created by matching them to the first time of bacterial infection, whereas the control group without bacterial infection was created by matching them in an age- and sex-related manner to the same timepoints of patients with a bacterial infection. The following virtual timepoints were created: the first measurement two days before the first bacterial infection (preV-2), the first measurement two days before the first bacterial infection (preV-1), the plasma level at the time of bacterial infection (V0) and the next measured plasma levels on the days afterwards (V1-V4). Data are presented as median with 95% Confidence interval as borders of the areas and single courses for each patient.

(b) Receiver operator characteristics (ROC)-analysis for bacterial IMX-BVN-3b scores in bacterial infected vs. uninfected patients regarding the indicated virtual timepoints as described above. Abbreviations: AUC, area under the curve; CI, confidence interval

Supplementary Figure 4: Viral IMX-BVN-3b scores in patients following liver transplantation.

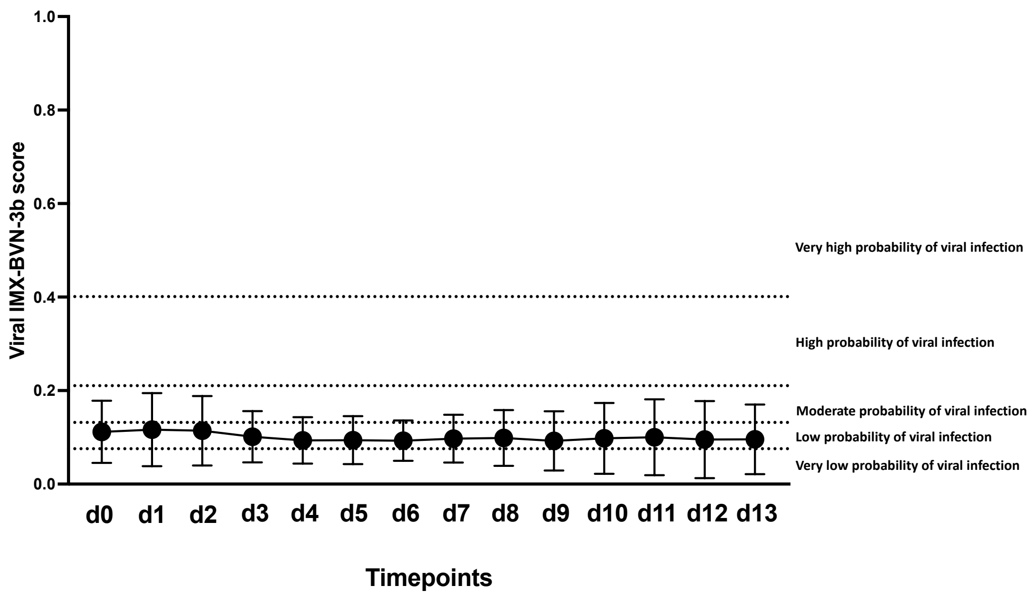

Viral IMX-BVN-3b scores were measured in patients following liver transplantation. Data in are given as the median with 95% confidence interval (CI).

Supplementary Table 1: Accuracy of IMX-BVN-3b, procalcitonin and C-reactive protein for the diagnosis of bacterial infections in 30 patients from day 0 to 13 after enrollment

Timepoint	IMX-BVN-3b Interpretation Band	Clinically Adjudicated Ground Truth		Sensitivity	Specificity	Likelihood ratio	Frequency of Results	Predictive Value
		Bacterial infection present	Bacterial infection absent					
day 0	Very Likely	7	15	0.7	0.25	0.93	0.73	0.32
	Likely	3	5	0.3	0.75	1.2	0.27	0.38
	Moderate	0	0	0	1	n.a	n.a	n.a
	Unlikely	0	0	n.a	n.a	n.a.	0	n.a
	Very Unlikely	0	0	n.a	n.a	n.a.	0	n.a
day 1	Very Likely	8	11	0.8	0.45	1.45	0.64	0.42
	Likely	2	8	0.2	0.6	0.5	0.33	0.20
	Moderate	0	1	0	0.95	0	0.03	0
	Unlikely	0	0	n.a	n.a	n.a.	0	n.a
	Very Unlikely	0	0	n.a	n.a	n.a.	0	n.a
day 2	Very Likely	7	9	0.7	0.55	1.56	0.54	0.44
	Likely	1	3	0.1	0.85	0.7	0.13	0.25
	Moderate	2	8	0.2	0.6	0.5	0.33	0.20
	Unlikely	0	0	n.a	n.a	n.a.	0	n.a
	Very Unlikely	0	0	n.a	n.a	n.a.	0	n.a
day 3	Very Likely	4	4	0.4	0.8	2	0.67	0.50
	Likely	5	5	0.5	0.75	2	0.33	0.50
	Moderate	1	7	0.1	0.65	0.28	0.27	0.13
	Unlikely	0	4	n.a	n.a	n.a.	0	n.a
	Very Unlikely	0	0	n.a	n.a	n.a.	0	n.a
day 4	Very Likely	5	2	0.5	0.9	5	0.23	0.71
	Likely	2	2	0.2	0.9	2	0.13	0.50
	Moderate	3	11	0.3	0.45	0.55	0.47	0.21
	Unlikely	0	5	1	0.25	0	0.17	0
	Very Unlikely	0	0	n.a	n.a	n.a.	0	n.a
day 5	Very Likely	4	1	0.4	0.95	8	0.17	0.8

	Likely	2	4	0.2	0.8	1	0.2	0.33
	Moderate	3	7	0.3	0.65	0.86	0.33	0.3
	Unlikely	1	8	0.83	0.4	0.40	0.3	0.11
	Very Unlikely	0	0	n.a	n.a	n.a.	0	n.a
day 6	Very Likely	4	1	0.4	0.95	8	0.17	0.8
	Likely	3	4	0.3	0.8	1.5	0.23	0.43
	Moderate	3	6	0.3	0.7	1	0.3	0.33
	Unlikely	0	8	1	0.42	0	0.27	0
	Very Unlikely	0	1	1	0.05	0	0.03	0
day 7	Very Likely	5	2	0.5	0.9	5	0.23	0.71
	Likely	2	3	0.2	0.85	1.33	0.17	0.40
	Moderate	3	8	0.3	0.6	0.75	0.37	0.27
	Unlikely	0	6	1	0.33	0	0.2	0
	Very Unlikely	0	1	1	0.06	0	0.03	0
day 8	Very Likely	5	1	0.5	0.95	10	0.2	0.83
	Likely	4	4	0.4	0.8	2	0.27	0.5
	Moderate	1	9	0.1	0.55	0.22	0.33	0.1
	Unlikely	0	6	1	0.32	0	0.2	0
	Very Unlikely	0	0	n.a	n.a	n.a.	0	n.a
day 9	Very Likely	6	1	0.6	0.95	12	0.23	0.86
	Likely	3	5	0.3	0.75	1.2	0.27	0.38
	Moderate	1	7	0.1	0.65	0.29	0.27	0.13
	Unlikely	0	7	1	0.37	0	0.23	0
	Very Unlikely	0	0	n.a	n.a	n.a.	0	n.a
T10	Very Likely	7	3	0.7	0.85	4.67	0.33	0.70
	Likely	0	2	0	0.9	0	0.07	0
	Moderate	3	8	0.3	0.6	0.75	0.37	0.27
	Unlikely	0	6	1	0.35	0	0.2	0
	Very Unlikely	0	1	1	0.06	0	0.03	0
T11	Very Likely	6	0	0.6	1	n.a	0.2	1
	Likely	1	6	0.1	0.7	0.33	0.23	0.14
	Moderate	3	5	0.3	0.75	1.2	0.27	0.38
	Unlikely	0	8	1	0.4	0	0.27	0
	Very Unlikely	0	1	1	0.05	0	0.03	0
T12	Very Likely	6	0	0.6	1	n.a	0.2	1

	Likely	1	3	0.1	0.85	0.67	0.13	0.25
	Moderate	2	9	0.2	0.55	0.44	0.37	0.18
	Unlikely	1	7	0.75	0.35	0.71	0.27	0.13
	Very Unlikely	0	1	1	0.05	0	0.03	0
T13	Very Likely	4	0	0.4	1	n.a	0.13	1
	Likely	2	5	0.2	0.75	0.8	0.23	0.29
	Moderate	3	2	0.3	0.9	3	0.17	0.6
	Unlikely	1	12	0.8	0.6	0.28	0.43	0.08
	Very Unlikely	0	1	1	0.05	0	0.03	0

B

Timepoint	PCT concentration	Ground Truth		Sensitivity	Specificity	Likelihood ratio	Frequency of Results	Predictive Value
		Bacterial infection	No bacterial infection					
day 0	> 0.5µg/L	9	17	0.9	0.15	1.06	0.87	0.35
	0.25-0.5µg/L	1	2	0.1	0.9	1	0.1	0.33
	0.1-0.25µg/L	0	0	1	0	n.a	0	n.a
	< 0.1µg/L	0	1	1	0.05	0	0.03	0
day 1	> 0.5µg/L	10	19	1	0.05	1.05	0.97	0.34
	0.25-0.5µg/L	0	1	0	0.95	0	0.03	0
	0.1-0.25µg/L	0	0	1	0	n.a	0	n.a
	< 0.1µg/L	0	0	1	0	n.a	0	n.a
day 2	> 0.5µg/L	10	19	1	0.05	1.05	0.97	0.34

	0.25-0.5µg/L	0	1	0	0.95	0	0.03	0
	0.1-0.25µg/L	0	0	1	0	n.a	0	n.a
	< 0.1µg/L	0	0	1	0	n.a	0	n.a
day 3	> 0.5µg/L	10	19	1	0.05	1.05	0.97	0.34
	0.25-0.5µg/L	0	0	0	1	n.a	0	n.a
	0.1-0.25µg/L	0	1	1	0.05	0	0.03	0
	< 0.1µg/L	0	0	1	0	n.a	0	n.a
day 4	> 0.5µg/L	10	19	1	0.05	1.05	0.97	0.34
	0.25-0.5µg/L	0	0	0	1	n.a	0	n.a
	0.1-0.25µg/L	0	1	1	0.05	0	0.03	0
	< 0.1µg/L	0	0	1	0	n.a	0	n.a
day 5	> 0.5µg/L	9	17	0.9	0.15	1.06	0.87	0.35
	0.25-0.5µg/L	0	3	0	0.85	0	0.1	0
	0.1-0.25µg/L	0	0	1	0	n.a	0	n.a
	< 0.1µg/L	1	0	0.9	0	n.a	0.03	1
day 6	> 0.5µg/L	10	14	1	0.3	1.43	0.8	0.42
	0.25-0.5µg/L	0	5	0	0.75	0	0.17	0
	0.1-0.25µg/L	0	0	1	0	n.a	0	n.a
	< 0.1µg/L	0	1	1	0.05	0	0.03	0
day 7	> 0.5µg/L	10	15	1	0.25	1.33	0.83	0.40
	0.25-0.5µg/L	0	3	0	0.85	0	0.1	0
	0.1-0.25µg/L	0	2	1	0.1	0	0.07	0
	< 0.1µg/L	0	0	1	0	n.a	0	n.a
day 8	> 0.5µg/L	7	15	0.70	0.25	0.9	0.73	0.32
	0.25-0.5µg/L	3	2	0.3	0.9	3	0.17	0.6
	0.1-0.25µg/L	0	3	1	0.15	0	0.1	0
	< 0.1µg/L	0	0	1	0	n.a	0	n.a
day 9	> 0.5µg/L	7	11	0.70	0.45	1.27	0.6	0.39
	0.25-0.5µg/L	2	6	0.2	0.70	0.67	0.27	0.25
	0.1-0.25µg/L	1	2	0.9	0.1	1	0.1	0.33
	< 0.1µg/L	0	1	1	0.05	0	0.03	0
day 10	> 0.5µg/L	7	8	0.70	0.6	1,75	0.5	0.47
	0.25-0.5µg/L	1	7	0.1	0.65	0.29	0.27	0.13

	0.1-0.25µg/L	2	4	0.8	0.2	1	0.2	0.33
	< 0.1µg/L	0	1	1	0.05	0	0.03	0
day 11	> 0.5µg/L	6	5	0.6	0.75	2.40	0.37	0.55
	0.25-0.5µg/L	2	8	0.2	0.6	0.5	0.33	0.2
	0.1-0.25µg/L	2	6	0.8	0.3	0.67	0.27	0.25
	< 0.1µg/L	0	1	1	0.05	0	0.03	0
day 12	> 0.5µg/L	6	4	0.6	0.8	3.	0.33	0.6
	0.25-0.5µg/L	2	4	0.2	0.8	1	0.2	0.33
	0.1-0.25µg/L	2	11	0.8	0.55	0.36	0.43	0.15
	< 0.1µg/L	0	1	1	0.05	0	0.03	0
day 13	> 0.5µg/L	6	4	0.6	0.8	3	0.33	0.6
	0.25-0.5µg/L	2	3	0.2	0.85	1.33	0.17	0.40
	0.1-0.25µg/L	0	12	1	0.6	0	0.40	0
	< 0.1µg/L	2	1	0.8	0.05	4	0.1	0.67

C

Timepoint	CRP concentration	Ground Truth		Sensitivity	Specificity	Likelihood ratio	Frequency of Results	Predictive Value
		Bacterial infection	No bacterial infection					
Pre day 0	> 80mg/L	2	1	0.2	0.95	4	0.1	0.67
	20-80mg/L	0	0	0	1	n.a	0	n.a
	10-20mg/L	1	2	0.9	0.1	1	0.1	0.33
	< 10mg/L	7	17	0.3	0.85	0.82	0.8	0.29
day 0	> 80mg/L	1	1	0.1	0.95	2	0.07	0.5
	20-80mg/L	0	0	0	1	n.a	0	n.a
	10-20mg/L	1	9	0.9	0.45	0.22	0.33	0.1
	< 10mg/L	8	10	0.2	0.5	1.6.	0.6	0.44
day 1	> 80mg/L	5	14	0.5	0.3	0.71	0.63	0.26
	20-80mg/L	0	0	0	1	n.a	0	n.a
	10-20mg/L	3	4	0.70	0.2	1.5	0.23	0.43

	< 10mg/L	2	2	0.8	0.1	2	0.13	0.5
day 2	> 80mg/L	6	8	0.6	0.6	1.5	0.47	0.43
	20-80mg/L	0	0	0	1	n.a	0	n.a
	10-20mg/L	3	10	0.70	0.5	0.6	0.43	0.23
	< 10mg/L	1	2	,0.9	0.1	1	0.1	0.33
day 3	> 80mg/L	6	5	0.6	0.75	2.4	0.37	0.55
	20-80mg/L	0	0	0	1	n.a	0	n.a
	10-20mg/L	2	7	0.8	0.35	0.57	0.3	0.22
	< 10mg/L	2	8	0.8	0.40	0.5	0.33	0.2
day 4	> 80mg/L	6	3	0.6	0.85	4	0.3	0.67
	20-80mg/L	0	0	0	1	n.a	0	n.a
	10-20mg/L	2	4	0.8	0.2	1	0.2	0.33
	< 10mg/L	2	13	0.8	0.65	0.31	0.5	0.13
day 5	> 80mg/L	6	3	0.6	0.85	4	0.3	0.67
	20-80mg/L	0	0	0	1	n.a	0	n.a
	10-20mg/L	2	5	0.8	0.25	0.8	0.23	0.29
	< 10mg/L	2	12	0.8	0.6	0.33	0.47	0.14
day 6	> 80mg/L	7	1	0.70	0.95	14	0.27	0.88
	20-80mg/L	0	0	0	1	n.a	0	n.a
	10-20mg/L	1	7	0.9	0.35	0.29	0.27	0.13
	< 10mg/L	2	12	0.8	0.6	0.33	0.47	0.14
day 7	> 80mg/L	8	5	0.8	0.75	3.0	0.43	0.62
	20-80mg/L	0	0	0	1	n.a	0	n.a
	10-20mg/L	0	4	1	0.2	0	0.13	0
	< 10mg/L	2	11	0.8	0.55	0.36	0.43	0.15
day 8	> 80mg/L	8	3	0.8	0.85	5.33	0.37	0.73
	20-80mg/L	0	0	0	1	n.a	0	n.a
	10-20mg/L	0	6	1	0.3	0	0.2	0
	< 10mg/L	2	11	0.8	0.55	0.36	0.43	0.15
day 9	> 80mg/L	8	3	0.8	0.85	5.33	0.37	0.73
	20-80mg/L	0	0	0	1	n.a	0	n.a
	10-20mg/L	1	6	0.9	0.3	0.33	0.23	0.14
	< 10mg/L	1	11	0.9	0.55	0.18	0.40	0.08
day 10	> 80mg/L	9	4	0.9	0.8	4.5	0.43	0.69
	20-80mg/L	0	0	0	1	n.a	0	n.a

	10-20mg/L	0	2	1	0.1	0	0.07	0
	< 10mg/L	1	14	0.9	0.70	0.14	0.5	0.07
day 11	> 80mg/L	9	4	0.9	0.8	4.5	0.43	0.69
	20-80mg/L	0	0	0	1	n.a	0	n.a
	10-20mg/L	0	3	1	0.15	0	0.1	0
	< 10mg/L	1	13	0.9	0.65	0.15	0.47	0.07
day 12	> 80mg/L	9	1	0.9	0.95	18	0.33	0.9
	20-80mg/L	0	0	0	1	n.a	0	n.a
	10-20mg/L	1	5	0.9	0.25	0.40	0.2	0.17
	< 10mg/L	0	14	1	0.70	0	0.47	0
day 13	> 80mg/L	9	3	0.9	0.85	6	0.40	0.75
	20-80mg/L	0	0	0	1	n.a	0	n.a
	10-20mg/L	1	4	0.9	0.2	0.5	0.17	0.2
	< 10mg/L	0	13	1	0.65	0	0.43	0

Legend: Performance of Bacterial IMX-BVN-3b after applying previously established cutoffs to segment scores into clinically actionable results interpretation bands according to [1]. Performance characteristics of the A: IMX-BVN-3b. B: procalcitonin (PCT) and C: C-reactive protein (CRP). When segmented into the results interpretation bands among patients with a proven bacterial infection. PCT and CRP were segmented into interpretation bands using cutoffs established in other studies [2-6]. Formulars are described in [7]. Abreviations: n.a.: not applicable

References:

1. He, Y.D.; Wohlford, E.M.; Uhle, F.; Buturovic, L.; Liesenfeld, O.; Sweeney, T.E. The Optimization and Biological Significance of a 29-Host-Immune-mRNA Panel for the Diagnosis of Acute Infections and Sepsis. *J Pers Med* **2021**, *11*, doi:10.3390/jpm11080735.
2. Chan, Y.L.; Liao, H.C.; Tsay, P.K.; Chang, S.S.; Chen, J.C.; Liaw, S.J. C-reactive protein as an indicator of bacterial infection of adult patients in the emergency department. *Chang Gung Med J* **2002**, *25*, 437-445.
3. Huang, D.T.; Yealy, D.M.; Filbin, M.R.; Brown, A.M.; Chang, C.H.; Doi, Y.; Donnino, M.W.; Fine, J.; Fine, M.J.; Fischer, M.A.; et al. Procalcitonin-Guided Use of Antibiotics for Lower Respiratory Tract Infection. *N Engl J Med* **2018**, *379*, 236-249, doi:10.1056/NEJMoa1802670.
4. Ip, M.; Rainer, T.H.; Lee, N.; Chan, C.; Chau, S.S.; Leung, W.; Leung, M.F.; Tam, T.K.; Antonio, G.E.; Lui, G.; et al. Value of serum procalcitonin, neopterin, and C-reactive protein in differentiating bacterial from viral etiologies in patients presenting with lower respiratory tract infections. *Diagn Microbiol Infect Dis* **2007**, *59*, 131-136, doi:10.1016/j.diagmicrobio.2007.04.019.
5. Rainer, T.H.; Chan, C.P.; Leung, M.F.; Leung, W.; Ip, M.; Lee, N.; Cautherley, G.W.; Graham, C.A.; Fuchs, D.; Renneberg, R. Diagnostic utility of CRP to neopterin ratio in patients with acute respiratory tract infections. *J Infect* **2009**, *58*, 123-130, doi:10.1016/j.jinf.2008.11.007.
6. Samsudin, I.; Vasikaran, S.D. Clinical Utility and Measurement of Procalcitonin. *Clin Biochem Rev* **2017**, *38*, 59-68.
7. Bauer, W.; Kappert, K.; Galtung, N.; Lehmann, D.; Wacker, J.; Cheng, H.K.; Liesenfeld, O.; Buturovic, L.; Luethy, R.; Sweeney, T.E.; et al. A Novel 29-Messenger RNA Host-Response Assay From Whole Blood Accurately Identifies Bacterial and Viral Infections in Patients Presenting to the Emergency Department With Suspected Infections: A Prospective Observational Study. *Crit Care Med* **2021**, *49*, 1664-1673, doi:10.1097/CCM.0000000000005119.

Supplementary Material 1: STROBE Statement—checklist of items that should be included in reports of observational studies

	Item No.	Recommendation	Page No.
**Title and abstract**	1	(*a*) Indicate the study's design with a commonly used term in the title or the abstract	1
		(*b*) Provide in the abstract an informative and balanced summary of what was done and what was found	4
**Introduction**			
Background/rationale	2	Explain the scientific background and rationale for the investigation being reported	8
Objectives	3	State specific objectives, including any prespecified hypotheses	8
**Methods**			
Study design	4	Present key elements of study design early in the paper	9
Setting	5	Describe the setting, locations, and relevant dates, including periods of recruitment, exposure, follow-up, and data collection	9-11
Participants	6	(*a*) *Cohort study*—Give the eligibility criteria, and the sources and methods of selection of participants. Describe methods of follow-up<br>*Case-control study*—Give the eligibility criteria, and the sources and methods of case ascertainment and control selection. Give the rationale for the choice of cases and controls<br>*Cross-sectional study*—Give the eligibility criteria, and the sources and methods of selection of participants	9-11
		(*b*) *Cohort study*—For matched studies, give matching criteria and number of exposed and unexposed<br>*Case-control study*—For matched studies, give matching criteria and the number of controls per case	
Variables	7	Clearly define all outcomes, exposures, predictors, potential confounders, and effect modifiers. Give diagnostic criteria, if applicable	9-11
Data sources/ measurement	8*	For each variable of interest, give sources of data and details of methods of assessment (measurement). Describe comparability of assessment methods if there is more than one group	9-11
Bias	9	Describe any efforts to address potential sources of bias	-
Study size	10	Explain how the study size was arrived at	-

Continued on next page

Quantitative variables	11	Explain how quantitative variables were handled in the analyses. If applicable, describe which groupings were chosen and why	11
Statistical methods	12	(*a*) Describe all statistical methods, including those used to control for confounding	11
		(*b*) Describe any methods used to examine subgroups and interactions	11
		(*c*) Explain how missing data were addressed	-
		(*d*) *Cohort study*—If applicable, explain how loss to follow-up was addressed<br>*Case-control study*—If applicable, explain how matching of cases and controls was addressed<br>*Cross-sectional study*—If applicable, describe analytical methods taking account of sampling strategy	11
		(*e*) Describe any sensitivity analyses	
**Results**			
Participants	13*	(a) Report numbers of individuals at each stage of study—eg numbers potentially eligible, examined for eligibility, confirmed eligible, included in the study, completing follow-up, and analysed	12-14
		(b) Give reasons for non-participation at each stage	-
		(c) Consider use of a flow diagram	-
Descriptive data	14*	(a) Give characteristics of study participants (eg demographic, clinical, social) and information on exposures and potential confounders	12-14
		(b) Indicate number of participants with missing data for each variable of interest	-
		(c) *Cohort study*—Summarise follow-up time (eg, average and total amount)	
Outcome data	15*	*Cohort study*—Report numbers of outcome events or summary measures over time	12-14
		Case-control study—Report numbers in each exposure category, or summary measures of exposure	
		Cross-sectional study—Report numbers of outcome events or summary measures	
Main results	16	(*a*) Give unadjusted estimates and, if applicable, confounder-adjusted estimates and their precision (eg, 95% confidence interval). Make clear which confounders were adjusted for and why they were included	Every figure
		(*b*) Report category boundaries when continuous variables were categorized	-
		(*c*) If relevant, consider translating estimates of relative risk into absolute risk for a meaningful time period	-

Continued on next page

Other analyses	17	Report other analyses done—eg analyses of subgroups and interactions, and sensitivity analyses	12-14
**Discussion**			
Key results	18	Summarise key results with reference to study objectives	15
Limitations	19	Discuss limitations of the study, taking into account sources of potential bias or imprecision. Discuss both direction and magnitude of any potential bias	17
Interpretation	20	Give a cautious overall interpretation of results considering objectives, limitations, multiplicity of analyses, results from similar studies, and other relevant evidence	15-18
Generalisability	21	Discuss the generalisability (external validity) of the study results	15-18
**Other information**			
Funding	22	Give the source of funding and the role of the funders for the present study and, if applicable, for the original study on which the present article is based	6

*Give information separately for cases and controls in case-control studies and, if applicable, for exposed and unexposed groups in cohort and cross-sectional studies.

Note: An Explanation and Elaboration article discusses each checklist item and gives methodological background and published examples of transparent reporting. The STROBE checklist is best used in conjunction with this article (freely available on the Web sites of PLoS Medicine at http://www.plosmedicine.org/, Annals of Internal Medicine at http://www.annals.org/, and Epidemiology at http://www.epidem.com/). Information on the STROBE Initiative is available at www.strobe-statement.org.
